# Endogenous Generation of Singlet Oxygen and Ozone in Human and Animal Tissues: Mechanisms, Biological Significance, and Influence of Dietary Components

**DOI:** 10.1155/2016/2398573

**Published:** 2016-03-06

**Authors:** Arnold N. Onyango

**Affiliations:** Department of Food Science and Technology, Jomo Kenyatta University of Agriculture and Technology, P.O. Box 62000, Nairobi 00200, Kenya

## Abstract

Recent studies have shown that exposing antibodies or amino acids to singlet oxygen results in the formation of ozone (or an ozone-like oxidant) and hydrogen peroxide and that human neutrophils produce both singlet oxygen and ozone during bacterial killing. There is also mounting evidence that endogenous singlet oxygen production may be a common occurrence in cells through various mechanisms. Thus, the ozone-producing combination of singlet oxygen and amino acids might be a common cellular occurrence. This paper reviews the potential pathways of formation of singlet oxygen and ozone in vivo and also proposes some new pathways for singlet oxygen formation. Physiological consequences of the endogenous formation of these oxidants in human tissues are discussed, as well as examples of how dietary factors may promote or inhibit their generation and activity.

## 1. Introduction

Singlet oxygen (^1^O_2_) is an electronically excited form of oxygen which is well known to be formed when photosensitizers such as chlorophyll or the aromatic dye rose bengal absorb light energy and transfer some of that energy to molecular oxygen [1, 2]. Various nonphotosensitized mechanisms for its formation have also been reported and suggested to occur in biological systems, but the importance of such endogenous singlet oxygen formation has had a controversial history [1, 3]. Ozone (O_3_) is best known as occurring in the stratosphere where it shields organisms on earth from ultraviolet C and much of ultraviolet B radiations, which are the most damaging UV components of solar radiations because they are readily absorbed by DNA [4, 5]. It is also known as a respiratory system-damaging pollutant in the troposphere and ironically as a therapeutic agent in alternative medicine [6]. More recently, it was shown that antibodies or amino acids catalyze the conversion of singlet oxygen (^1^O_2_) to ozone (O_3_) and that this reaction occurs during the killing of bacteria by activated neutrophils [7, 8]. Since both singlet oxygen and ozone are highly reactive oxygen species, a full understanding of their mechanisms of formation and action in vivo is necessary. Hence, this paper reviews the various reported mechanisms of the endogenous formation of these reactive oxygen species (ROS), the potential relevance of such pathways in human physiology, and how dietary factors affect the generation and activity of these oxidants.

## 2. Radiation-Induced Formation of Singlet Oxygen

Human beings are frequently exposed to natural and artificial radiation, and most of this interacts primarily with the skin. The spectrum of solar radiation at the earth's surface consists of ultraviolet (UV) radiation (UVB: 290–320 nm and UVA: 320–400 nm), visible radiation (VIS: 400–760 nm), and near infrared radiation (IRA: 760–1440 nm and IRB: 1440–3000 nm) [9]. UV, VIS, and IR contribute 7%, 39%, and 54% of the solar energy reaching the skin [10]. Direct absorption of UVB by cellular DNA leads to formation of cyclobutane pyrimidine dimers and pyrimidine (6–4) pyrimidone products, while UVA is not readily absorbed by DNA, and its direct damage to DNA is therefore not important [5]. Nevertheless, both UVA and UVB as well as visible light convert various photosensitizing compounds to excited states which transfer energy to triplet oxygen, thereby generating reactive oxygen species, particularly singlet oxygen.

UVA makes up 95% of the UV reaching the human skin, and up to 50% of it can penetrate to the dermis, unlike UVB that only penetrates the epidermis [11]. The human skin is rich in UVA and visible light (particularly the blue region) photosensitizers such as porphyrins, bilirubin, flavins, melanin and melanin precursors, pterins, B_6_ vitamers, and vitamin K [12, 13]. The formation of singlet oxygen in the skin as a result of the interaction of UVA with these photosensitizers has been demonstrated directly by luminescence [14] and by detection of cholesterol-5-hydroperoxide which is preferentially generated by singlet oxygen but not by free radical mediated cholesterol oxidation [2]. The interaction between UVB and various vitamins and fatty acids also results in the generation of singlet oxygen, and some compounds including vitamin E that are ordinarily not UVA photosensitizers can be converted to UVA photosensitizers if they are preirradiated with UVB [11]. Photosensitized formation of singlet oxygen also occurs in the retina, which contains endogenous photosensitizers and is exposed to light [15]. One of the singlet oxygen-generating photosensitizers is lipofuscin, which forms in the retinal pigment epithelium with age or genetic disorders such as Stargardt's disease [15, 16]. Ground state oxygen can directly absorb visible light of 765 nm, even in mammalian cells, leading to formation of singlet oxygen without the involvement of a photosensitizer [17]. Similarly, IRB of 1268 nm can cause direct conversion of ground state oxygen to singlet oxygen [18].

Both IRB and IRC penetrate the skin only shallowly, while IRA (which makes up 30% of the total IR radiation reaching the skin) penetrates deeply, with 65% of it reaching the dermis [19, 20]. Unlike UVA, IRA penetration of the skin does not cause photosensitized formation of singlet oxygen but initiates the formation of reactive oxygen species, mainly from the mitochondrial electron transport chain [9, 20, 21]. While singlet oxygen may be one of these ROS [9], its specific detection under such circumstances has not been studied. However, both UVA and IR induce upregulation of matrix metalloproteinases (MMPs) and thereby promote photoaging [9, 19]. The UV-induced MMP expression is dependent on cholesterol-5 hydroperoxide, a product of oxidation of cholesterol by singlet oxygen [2, 22]. Whether IR-induced metalloproteinase activation also depends to a great extent on singlet oxygen and cholesterol-5 hydroperoxide remains to be demonstrated. In this case, the role of IR in singlet oxygen formation may simply involve initiating the formation of superoxide anions, from which singlet oxygen would be generated by various types of radiation-independent reactions (vide infra). Singlet oxygen formation in organs other than the skin and eye mainly depends on such “dark” reactions.

Artificial sources of radiation may also contribute to endogenous singlet oxygen formation in humans. For example, during photodynamic therapy, a photosensitizer is inserted into cancerous tissue and irradiated with UV to produce singlet oxygen which serves the purpose of destroying cancer cells [23]. IR irradiation is commonly used in medicine to warm muscle tissue [24] and might also contribute to singlet oxygen formation.

## 3. Leukocyte-Mediated Formation of Singlet Oxygen

Neutrophils, including human neutrophils, produce singlet oxygen [7, 35–37] and this has been suggested to be important for bacterial killing through the formation of ozone [7]. It is generally considered that production of singlet oxygen by neutrophils is dependent on myeloperoxidase (MPO) which catalyzes the formation of hypochlorous acid (HOCl) from hydrogen peroxide (H_2_O_2_) and chloride ion (see equation (1)), followed by reaction of HOCl with hydrogen peroxide anion (HO_2_^−^) (see equation (2)) [7, 37]. However, the significance of the reaction between HO_2_^−^ and HOCl under physiological environments such as the intraphagosomal milieu may be limited by the presence of other reactive partners for HOCl [36], and it was suggested that alternative pathways of singlet oxygen generation by neutrophils may exist, including the spontaneous dismutation of superoxide anions (see equation (3)) [36, 37]. However, the yield of singlet oxygen from the latter reaction was also found to be minor [38]. Peritoneal macrophages, which are MPO deficient, produce higher yield of singlet oxygen than neutrophils [37]. In the macrophage phagosome, the reaction between nitric oxide (NO^∙^) and superoxide anion (^∙−^O_2_) occurs at diffusion-controlled rates to form peroxynitrite (ONOO^−^) (see equation (4)) [39], which reacts with H_2_O_2_ to produce singlet oxygen (see equation (5)) [40]. The reaction of NO^∙^ with H_2_O_2_ was also found to generate singlet oxygen in a purely chemical system and in a superoxide generating system (see equation (6)) [41, 42]. The eosinophil peroxidase system generates singlet oxygen by a reaction between HOBr and HO_2_^−^, analogously to (2) [30]:

(1) H2O2+Cl−+H+⟶HOCl+H2O

(2) HOCl+HO2−⟶H2O+Cl−+O21

(3) O2∙−+O2∙−+2H+⟶O21+H2O2

(4) O2∙−+NO∙⟶ONOO−

(5) ONOO−+H2O2⟶ONO−+H2O+O21

(6) O2∙−+H2O2⟶O21+HO∙+HO−

(7) H2O+O21⟶H2O3→O21H2O2+O3

## 4. Singlet Oxygen Formation by the Russel Mechanism

**Figure 1:**
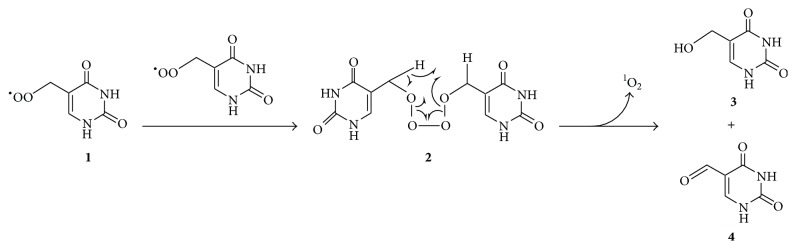
Formation of ^1^O_2_ through reaction of thymine peroxyl radicals (**1**) by the Russell mechanism [25].

Russell [43] proposed the idea that two peroxyl radicals can react to form an unstable tetroxide whose decomposition affords singlet oxygen, an alcohol, and a carbonyl compound, and this mechanism is now believed to contribute to singlet oxygen formation from various biomolecules including proteins, lipids, and nucleic acids [44]. The oxidation of DNA was found to result in singlet oxygen by this mechanism as illustrated in Figure 1, whereby thymine peroxyl radicals** 1** react to generate tetroxide** 2** whose decomposition produces alcohol** 3**, carbonyl** 4**, and ^1^O_2_ [25].

**Figure 2:**
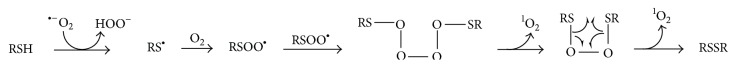
Russell-type mechanism for formation of ^1^O_2_ from the reaction of glutathione (RSH) with superoxide anions (^∙−^O_2_) [26].

Cysteine residues in glutathione (RSH) were found to be readily oxidized by superoxide anions to form singlet oxygen, glutathione disulfide (RSSR), and glutathione sulfonate (RSO_3_^−^) in a reaction that was suggested to involve the peroxysulphenyl radical (RSOO^∙^) [26] and can be considered as a special type of Russel mechanism (Figure 2). This mechanism may also apply to cysteine residues in proteins. Hydroxyl radicals may also initiate the conversion of amino acids to peroxyl radicals which then participate in the Russell mechanism [44].

As reviewed by Miyamoto et al. [45], various studies have demonstrated the formation of singlet oxygen by the Russel mechanism during the decomposition of lipid hydroperoxides (ROOH) in the presence of species such as Fe^3+^, Cu^2+^, peroxynitrite, HOCl, or cytochrome c, which oxidize the hydroperoxides to the corresponding peroxyl radicals (ROO^∙^). However, while singlet oxygen formation by the Russell mechanism in such purely chemical systems is established, its importance in tissues has been considered debatable because high concentrations of peroxyl radicals are unlikely to develop under such systems [46, 47]. On the other hand, the fact that peroxyl radicals derived from phospholipid and cholesterol hydroperoxides in liposomes produced singlet oxygen was considered as an indication that this phenomenon may occur in cellular membranes [45]. Moreover, cytochrome c was found to promote oxidation of polyunsaturated fatty acid-containing cardiolipin, with concomitant singlet oxygen formation, and this may be relevant in the mitochondria where both species exist [45].

**Figure 3:**
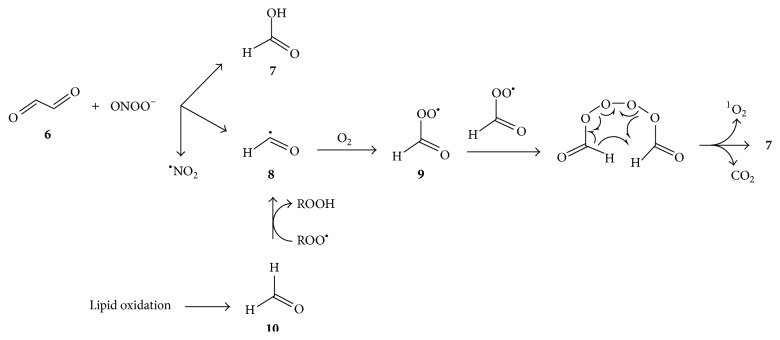
Russell-type mechanism for formation of ^1^O_2_ upon reaction of glyoxal (**6**) with peroxynitrite (ONOO^−^) via formyl radical (**8**) [27] and proposed occurrence of such a reaction via formyl radicals formed during lipid oxidation.

Peroxynitrite (ONOO^−^) reacts with glyoxal to produce singlet oxygen, and this was proposed to involve cleavage of glyoxal** 6** to formic acid** 7** and formyl radical** 8**, with subsequent conversion of the latter to peroxyformyl radical** 9**, and reaction of two such peroxyacyl radicals by the Russel mechanism as shown in Figure 3 [27]. Even in the absence of peroxynitrite, aldehydes formed during lipid autoxidation are easily converted to the corresponding acids via acyl and peroxyacyl radicals [48], and these may similarly produce singlet oxygen, as illustrated for the conversion of lipid oxidation-derived formaldehyde** 10** to formic acid and singlet oxygen (Figure 3).

## 5. Singlet Oxygen Formation via the Dismutation of Alkoxyl Radicals

**Figure 4:**
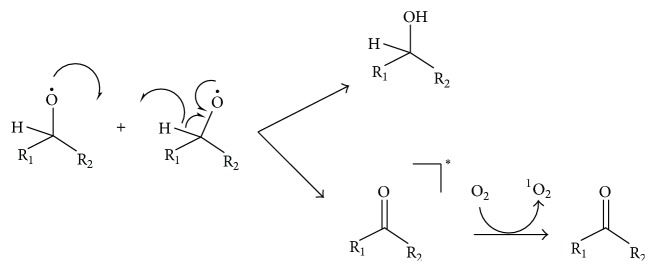
Formation of ^1^O_2_ via dismutation of alkoxyl radicals [28]. The asterisk indicates that the carbonyl is in excited state.

Two alkoxyl radicals (RO^∙^) can undergo dismutation to form a carbonyl and an alcohol (Figure 4), and some of the carbonyls are formed in the excited triplet state, with a yield of up to 8% [28]. The triplet carbonyls can transfer energy to triplet oxygen, thereby generating singlet oxygen [28]. Because alkoxyl radicals are major intermediates during decomposition of biological hydroperoxides [28, 48], the potential contribution of this pathway to singlet oxygen formation cannot be ignored.

## 6. Singlet Oxygen Formation via the Oxidation of Phenolic Substances

**Figure 5:**
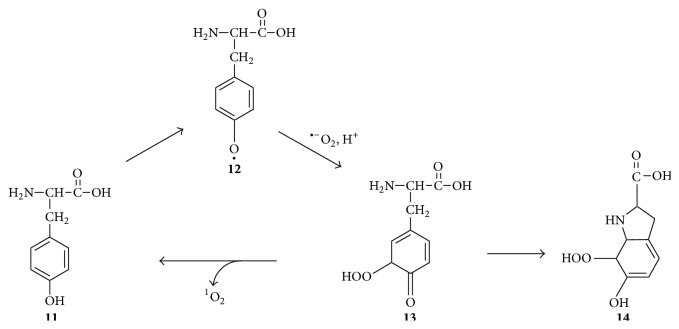
Formation of ^1^O_2_ during the superoxide-dependent oxidation of tyrosine** 11** [29].

Phenolic substances are important components of the human diet, and one of such compounds is the amino acid tyrosine. In many physiological situations, tyrosine** 11** gets converted to the tyrosyl radical** 12**, which in turn gets converted by superoxide anions to tyrosine hydroperoxide** 13**, whose decomposition may produce singlet oxygen and regenerate tyrosine (Figure 5) [40]. However, tyrosine hydroperoxide** 13** also gets converted to its bicyclic isomer** 14**, whose decomposition does not produce singlet oxygen, and this greatly reduces the amount of singlet oxygen formed from this system [29]. Nevertheless, this mechanism may be important because tyrosine and tryptophan residues are known to be major contributors to protein-dependent singlet oxygen formation [32].

**Figure 6:**
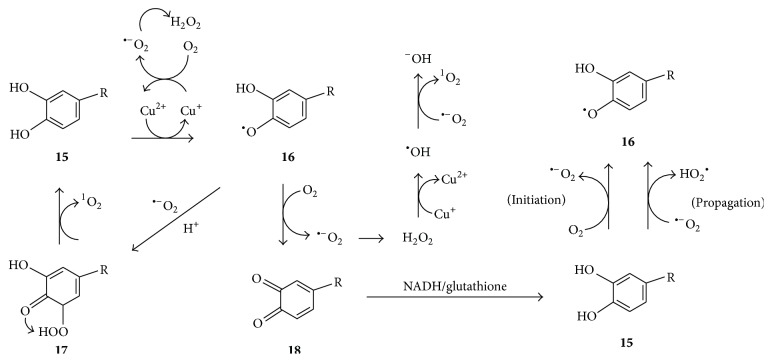
Proposed formation of ^1^O_2_ from catechol derivative** 15** via hydroperoxide** 17** formed by superoxide anion addition and ^1^O_2_ formation via oxidation of semiquinone radical** 16** to quinone** 18** in the presence of Cu^2+^ ions [30]. Catechol derivative** 15** may undergo oxidation to the semiquinone radical** 16** without metal catalysis [31].

It is reasonable to expect that singlet oxygen will be formed from other phenolic substances analogously to its formation from tyrosine according to Figure 5. For example, Figure 6 illustrates singlet oxygen formation from catechol or a catechol derivative** 15** via a semiquinone radical** 16** and a hydroperoxy semiquinone** 17**. Consistent with this, the 2-hydroxycatechol of estradiol was found to be oxidized by Cu^2+^ ions to produce superoxide ion, hydrogen peroxide, and singlet oxygen, and its DNA-strand breaking activity was diminished by singlet oxygen quenchers but not by hydroxyl radical quenchers [52]. Similarly, the substantial formation of singlet oxygen during Cu^2+^-mediated oxidation of catecholamines, derivatives of catechol, was reported by Kruk et al. [53]. These authors proposed the following sequence of events to be involved in singlet oxygen formation: (i) Cu^2+^ oxidizes the catecholamine (represented by** 15** in Figure 6) to the semiquinone radical** 16**; (ii) Cu^+^ generated in the process reduces O_2_ to superoxide anion (^∙−^O_2_) and triplet oxygen may also oxidize** 16** to quinone** 18**, with formation of ^∙−^O_2_; (iii) dismutation of the latter affords H_2_O_2_; (iv) Cu^+^ reduces H_2_O_2_ to hydroxyl radicals (^∙^OH) whose reaction with ^∙−^O_2_ affords singlet oxygen (Figure 6).

Akagawa et al. [31] further found that at 37°C and pH 7.4 various phenolic substances such as pyrogallol, pyrocatechol, 1,2,4-benzenetriol, and catechin, as well as polyphenol rich extracts of tea and coffee, generated significant amounts of H_2_O_2_ in both the presence and the absence of metal ions. Thus, they suggested a metal ion-free mechanism for generation of the semiquinone radical** 16**, consisting of an initiation step involving a noncatalyzed oxidation of the phenolic substance** 15** by oxygen to form superoxide anion, followed by a propagation stage involving oxidation of the phenolic substance by superoxide anion as illustrated in Figure 6. The generation of superoxide anion and hydrogen peroxide is not unique to polyphenols, since even ascorbic acid undergoes such a reaction [54]. Tyihák et al. [6] reported that the antimicrobial activity of phenolic substances such as cinnamic acid and transresveratrol depends on their generation of singlet oxygen, and this may involve reactions as depicted in Figure 6.

## 7. Singlet Oxygen Formation via Dioxetanes

Dioxetanes are high energy 1,2-peroxides whose decomposition affords excited carbonyls in high yields [32]. Rác et al. [47] recently suggested that their observed formation of singlet oxygen in U937 human leukemic cells treated with H_2_O_2_ or the Fenton reagent was mainly due to decomposition of dioxetane intermediates.

**Figure 7:**
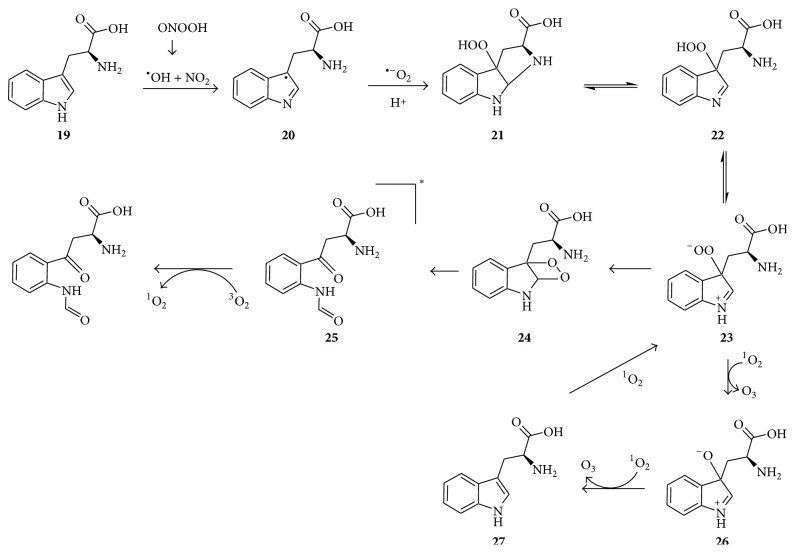
Formation of ^1^O_2_ during the oxidation of tryptophan** 19** via dioxetane** 24** [32, 33] and formation of O_3_ by ^1^O_2_-mediated deoxidation of intermediates** 23** and** 26** [34]. The asterisk indicates that the carbonyl is in excited state.

Singlet oxygen formation during protein oxidation to a large extent involves tryptophan and tyrosine residues, and the chemiluminescence from tryptophan has long been regarded to potentially involve a dioxetane intermediate whose decomposition affords N-formylkynurenine [32]. Michalski et al. [33] recently reported on the peroxynitrite and superoxide-mediated conversion of tryptophan** 19** via radical** 20** to bicyclic tryptophan hydroperoxide** 21** (Figure 7). Hydroperoxide** 21** is also a known product of the singlet oxygen-mediated oxidation of tryptophan, and it gets converted via its hydroperoxide isomer** 22**, zwitterion** 23**, and dioxetane** 24** to N-formylkynurenine** 25** [34, 55]. The latter has a high chance of being formed in a triplet state, which will transfer energy to O_2_ and generate ^1^O_2_. As explained later, hydroperoxide** 23** may also participate in ozone-generating reactions via intermediates** 26** and** 27**.

**Figure 8:**
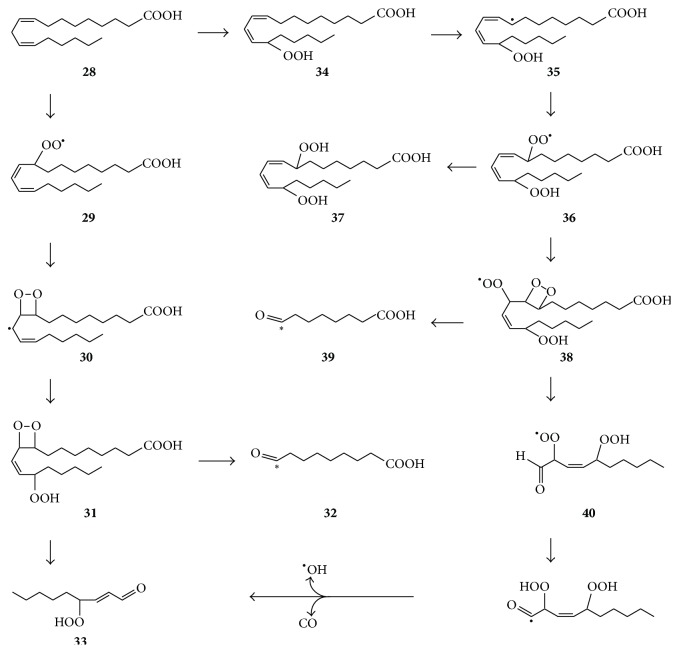
Postulated conversion of linoleic acid** 28** via dioxetanyl derivative** 31** to major products 9-oxononanoic acid** 32** and 4-hydroperoxy-2-nonenal** 33** [49] or via dioxetanyl radical** 38** to hydroperoxyaldehyde** 33** and 8-oxooctanoic acid** 39** [50]. Decomposition of the dioxetanyl derivatives affords excited carbonyls that transfer energy to O_2_, thus forming ^1^O_2_. The asterisk indicates that the carbonyl is in excited state.

The formation of some fatty acid oxidation products may be rationalized by pathways involving dioxetanyl derivatives formed by cyclization of peroxyl radicals. The cyclization of a model peroxyl radical derived from 3-hydroperoxy-2,3-dimethyl-1-butene (TMEOOH), and its subsequent conversion to excited carbonyls via a dioxetanyl intermediate was demonstrated by Timmins et al. [56]. Kaur et al. [49] postulated that such peroxycylization was an important pathway for the formation of major aldehydic products of linoleic acid oxidation, such as 9-oxononanoic acid and 4-hydroperoxy-2-nonenal. The latter is a precursor of the highly reactive and biologically active products, 4-hydroxy-2-nonenal and 4-oxo-2-nonenal, hence the great interest in its mechanism of formation [49, 50, 56–58]. It was proposed that linoleic acid** 28** gets converted to peroxyl radical** 29**, whose cyclization affords dioxetanyl radical** 30** as a precursor of hydroperoxy dioxetane** 31**, whose decomposition affords 9-oxononanoic acid** 32** and 4-hydroperoxy-2-nonenal** 33** (Figure 8) [50]. One of the latter aldehydes may be in the triplet state and thus may be a source of energy for conversion of triplet oxygen to singlet oxygen. Lee et al. [57] found that the 13-hydroperoxide of linoleic acid (13-LA-OOH,** 34**) was a major precursor of aldehydes** 32** and** 33**, with retention of the -OOH group of** 34** in** 33**. Schneider et al. [58] further found that, during conversion of 13-LA-OOH** 34** to aldehydes** 32** and** 33**, there was facile conversion of hydroperoxide** 34** via radicals** 35** and** 36** to its 8,13-dihydroperoxy-derivative** 37** (Figure 8) and 8-oxooctanoic acid was also formed. This is consistent with another postulated pathway for the formation of hydroperoxyaldehyde** 33** involving cyclization of peroxyl radical** 36** to form dioxetanyl derivative** 38**, whose decomposition affords 8-oxooctanoic acid** 39** and radical** 40**, a precursor** 33** [50].

**Figure 9:**
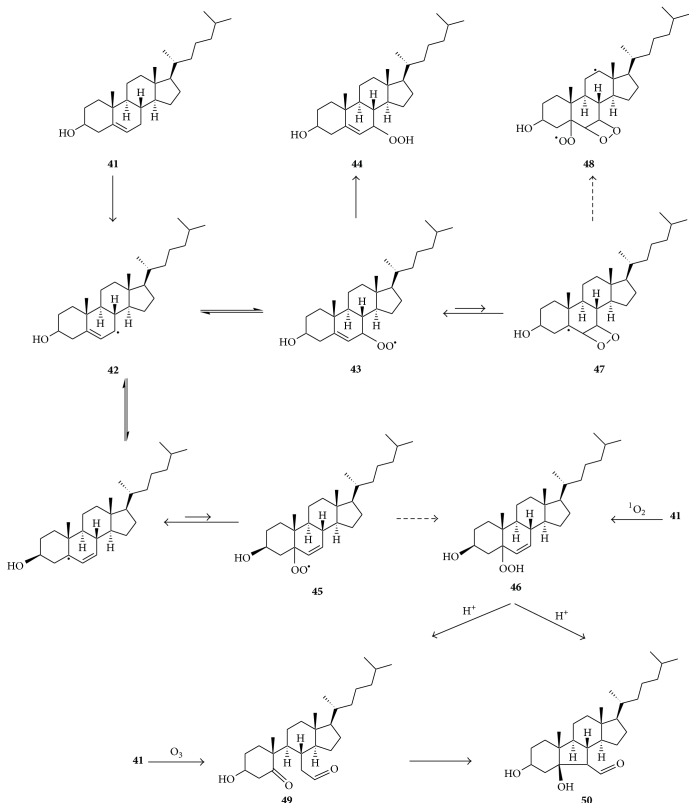
Oxidation pathways of cholesterol** 41**, leading to the 7-hydroperoxide** 44** during autoxidation, the 5-hydroperoxide** 46** upon reaction with ^1^O_2_, and secosterol A (**49**) upon reaction with O_3_. Aldolization of** 49** produces secosterol B (**50**). Hock cleavage of** 46** predominantly produces secosterol** 50** and minor amounts of** 49**. There is no evidence of formation of dioxetanyl derivatives.

On the other hand, there is no evidence for the formation of dioxetanyl derivatives during cholesterol oxidation (Figure 9).

The autoxidation of cholesterol** 41** proceeds via carbon-centered radical** 42** and peroxyl radical** 43** to generate cholesterol-7-hydroperoxide** 44** as a major product [59, 60]. While carbon-centered radical** 42** might also be expected to isomerize and subsequently be converted via peroxyl radical** 45** to cholesterol 5-hydroperoxide** 46**, formation of the latter during cholesterol autoxidation is negligible, even though** 46** is the major product of cholesterol oxidation by singlet oxygen [59, 60]. Lack of formation of** 46** during autoxidation has been attributed to a fast rate of dissociation of oxygen from peroxyl radical** 45** [60], indicating higher stability of radical** 43** than** 45**. Although peroxyl radical** 43** is easily converted to hydroperoxide** 44**, there is no evidence that the former undergoes cyclization to form dioxetanyl derivatives** 47** and** 48**, because aldehydic products expected from the decomposition of the latter two have not been reported. This might likewise be due to a much higher stability of radical** 43** than radicals** 47** and** 48**. Ozone directly converts cholesterol** 41** to the secosterol aldehyde** 49** (secosterol A), which undergoes some conversion to its aldolization product** 50** (secosterol B) [61]. Hock cleavage of cholesterol 5-hydroperoxide** 46** under acidic conditions affords mainly secosterol B** 50** with minor amounts of secosterol A** 49** [61]. However, no analogous C-C cleavage products attributable to decomposition of dioxetanyl derivatives arising from cholesterol peroxyl radical cyclizations are known. Thus, decomposition of cholesterol hydroperoxides by the Russell mechanism [62] may be the only major pathway for singlet oxygen generation from cholesterol.

## 8. Singlet Oxygen Formation by the Reaction of Superoxide Anion with Hydrogen Peroxide

The reaction of superoxide anion with hydrogen peroxide to form singlet oxygen, hydroxyl radical, and hydroxide ion (see equation (6)), a modified form of the Haber-Weiss reaction, was proposed by Kellogg and Fridovich [63] and demonstrated upon the reaction of potassium superoxide with hydrogen peroxide in a simple reaction system [64]. However, this reaction is controversial: Koppenol [65] registered strong disapproval for it, mainly based on the fact that various studies found that the rate constant for the Haber-Weiss reaction is in the order of 1 M^−1^ s^−1^ or less.

## 9. Singlet Oxygen Formation via Cytochrome c-Mediated Formation of Triplet Carbonyls

Cytochrome c converts carbonyls such as lipid-derived aldehydes to triplet carbonyls, which then transfer energy to oxygen, thus generating singlet oxygen [66]. In fact, singlet oxygen formation from a model membrane having polyunsaturated fatty acid-containing cardiolipin in association with cytochrome C was found to be more dependent on triplet carbonyls than on the decomposition of hydroperoxides via the Russel mechanism [66].

## 10. Singlet Oxygen Formation by the Reaction of Hydroperoxides with Carbonyls

Under certain conditions such as in the presence of pyrogallol, lysine, tryptophan, or superoxide anions, the interaction of H_2_O_2_ with carbonyls such as formaldehyde, acetaldehyde, glyoxal, methyl-glyoxal, and even glucose was demonstrated to produce singlet oxygen and reactive aldehydes [51, 67–72], and such conditions should be common in vivo: considering that carbonyls are major lipid oxidation and glycoxidation products, all cells have formaldehyde generating pathways referred to as the formaldehydome [6, 69, 71], and hydrogen peroxide is also generated through many enzymatic and nonenzymatic reactions. The biological relevance of the reaction of H_2_O_2_ with carbonyls has been demonstrated in several studies. For example, brewed coffee and instant coffee give strong chemiluminescence due to singlet oxygen and reactive aldehydes [73], and the mutagenicity of coffee has been partly attributed to its content of both methylglyoxal and hydrogen peroxide [74]. The mutagenicity of glyoxal in* Salmonella* was found to be dependent on singlet oxygen generation and that catalase or scavengers of H_2_O_2_ reduced the mutagenic effect [75]. Kim et al. [76] found mixtures of glucose and lysine or arginine to be mutagenic and that such activity greatly depended on the formation of hydrogen peroxide and singlet oxygen. Maillard reaction products prepared by heating equimolar mixtures of glucose and amino acids, when incubated with DNA under physiological conditions, were reported to have DNA-strand breaking activity accompanied by singlet oxygen formation [77].

**Figure 10:**
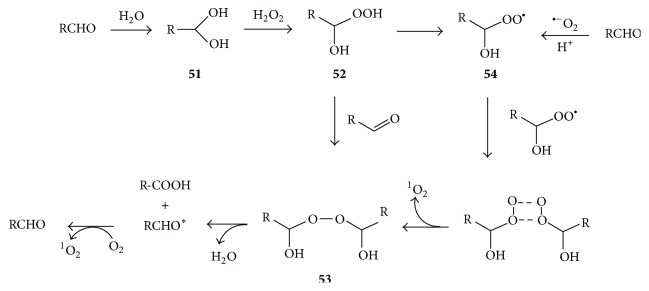
Mechanism of formation of ^1^O_2_ and triplet carbonyls during reaction of H_2_O_2_ with carbonyls [51]. The asterisk indicates that the carbonyl is in excited state.

Hydroperoxides are very good nucleophiles because of the alpha effect, whereby interaction of lone electron pairs on two adjacent oxygen atoms increases nucleophilicity [78], and this explains the reactivity of hydrogen peroxide with aldehydes. Trézl and Pipek [51] proposed the pathways illustrated in Figure 10 for the generation of singlet oxygen during such H_2_O_2_-carbonyl reactions. First, the facile hydration of an aldehyde (RCHO) produces a gem diol** 51** which reacts with H_2_O_2_ to form a 1-hydroxyalkylhydroperoxide** 52**. The latter may react with another molecule of aldehyde to form bis-1-hydroxyalkylperoxide** 53** whose decomposition affords an acid and an excited aldehyde which then participates in singlet oxygen formation. Alternatively the 1-hydroxyalkylhydroperoxide** 52** is oxidized to form a 1-hydroxyalkylperoxyl radical** 54** that undergoes Russel-type decomposition. The oxidation of formaldehyde CH_2_O by H_2_O_2_ in the presence of pyrogallol is called the Trautz-Schorigin reaction, a very efficient source of singlet oxygen whereby the semiquinone radical derived from pyrogallol is responsible for oxidizing the 1-hydroxyalkylhydroperoxide** 52** to form** 54** [79]. Superoxide anion also enhances formation of** 54** [79].

**Figure 11:**
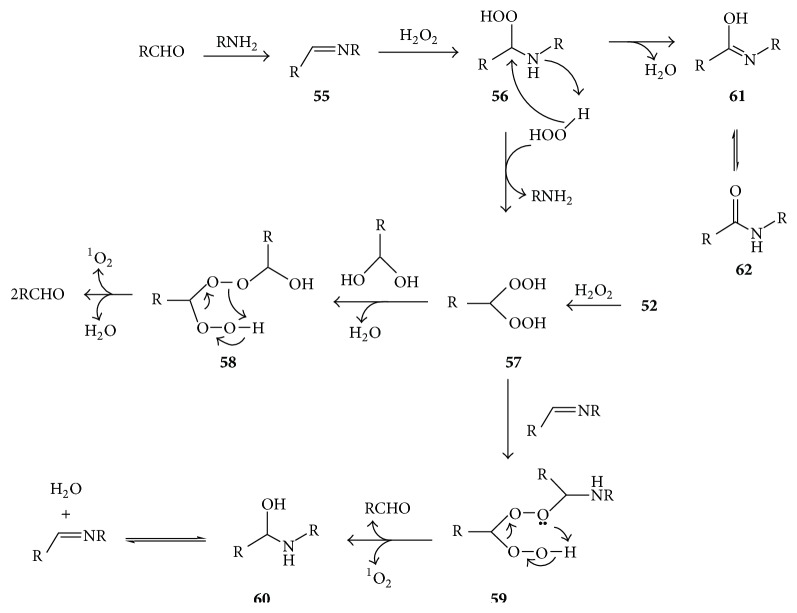
Proposed mechanisms of lysine- (RNH_2_-) promoted reaction of H_2_O_2_ with carbonyls to generate ^1^O_2_ and the formation of carbonylated lysine products (**62**) under such conditions.

The mechanism involved in the lysine or tryptophan-catalyzed formation of singlet oxygen from hydrogen peroxide and aldehyde has not been clearly defined. However, it is conceivable that this involves the pathways suggested in Figure 11, which is based on several known reactions.

First, the carbonyl (RCHO) reacts with the lysine (RNH_2_) to form Schiff's base** 55**, which then adds H_2_O_2_ to form hydroperoxide** 56**. The latter reacts with another H_2_O_2_ molecule, resulting in regeneration of lysine and formation of a 1,1-dihydroperoxide** 57**. Gem dihydroperoxide** 57** may also be formed in an uncatalyzed reaction between H_2_O_2_ and 1-hydroxyhydroperoxide** 52** [80, 81]. Hang et al. [82] reported that some monosubstituted 1,1-dihydroperoxides undergo decomposition to produce singlet oxygen in high yield. A reaction of** 57** with a gem diol may generate such monosubstituted 1,1-dihydroperoxide** 58** as a precursor of singlet oxygen. A reaction of** 57** with Schiff's base may similarly lead to singlet oxygen formation via monosubstituted 1,1-dihydroperoxide** 59**.

**Figure 12:**
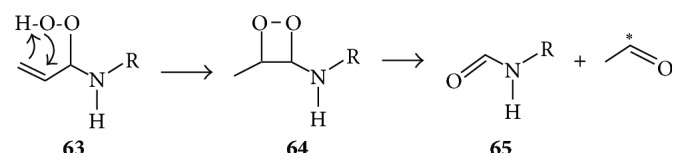
Proposed mechanism for conversion of acrolein-derived hydroperoxide** 63** to formyl lysine** 65** and acetaldehyde. The asterisk indicates that the carbonyl is in excited state.

The reaction of H_2_O_2_ with formaldehyde or acrolein in the presence of lysine also leads to the formation of formyl lysine while acetaldehyde generates acetyl lysine [68]. Dehydration of hydroperoxide** 56** may lead to such carbonylated lysine products represented by isomeric structures** 61** and** 62** (this involves a hydride transfer from carbon to oxygen in** 56**). The fact that, like formaldehyde, acrolein (2-propenal) generates formyl lysine may be explained by the reaction illustrated in Figure 12, whereby the acrolein-derived hydroperoxide** 63** rearranges to a dioxetane intermediate** 64** whose decomposition affords formyl lysine** 65** and a triplet acetaldehyde which may also be a source of energy for singlet oxygen production.

**Figure 13:**
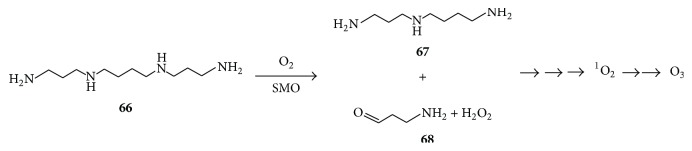
Spermine oxidase- (SMO-) catalyzed catabolism of spermine** 66** to form H_2_O_2_, spermidine** 67**, and 3-aminopropanal** 68**, which can participate in further reactions leading to ^1^O_2_ and O_3_ formation.

Eukaryotic cells synthesize polyamines such as spermine and spermidine, which are essential for normal cell growth and development [83, 84]. These compounds are catabolized by polyamine oxidases such as spermine oxidase (SMO) which catalyzes the conversion of spermine** 66** to spermidine** 67**, H_2_O_2_, and 3-aminopropanal** 68** (Figure 13) [83–85].

The coformation of these three products creates an ideal situation for singlet oxygen according to Figure 10 or Figure 11, since both aminopropanal and spermidine contain the amino group, like lysine. This might be a key aspect in the mechanism of the known polyamine-dependent development of cancers such as gastric cancer [83, 84]. A recent study reported that Cu (II) polypyridyl complexes reduced the growth of breast cancer cells, and it was suggested that this was partly due to the production of singlet oxygen or a singlet oxygen-like compound that cleaved supercoiled DNA [86]. The expression of SMO in these cells also reduces their growth [87], which could likewise be due to singlet oxygen generation.

Neutrophils employ myeloperoxidase to oxidize nearly all amino acids found in plasma to aldehydes in high yield [88]. The reactions of these aldehydes with neutrophil-generated hydrogen peroxide may thus be another important mechanism for singlet oxygen production by neutrophils.

**Figure 14:**
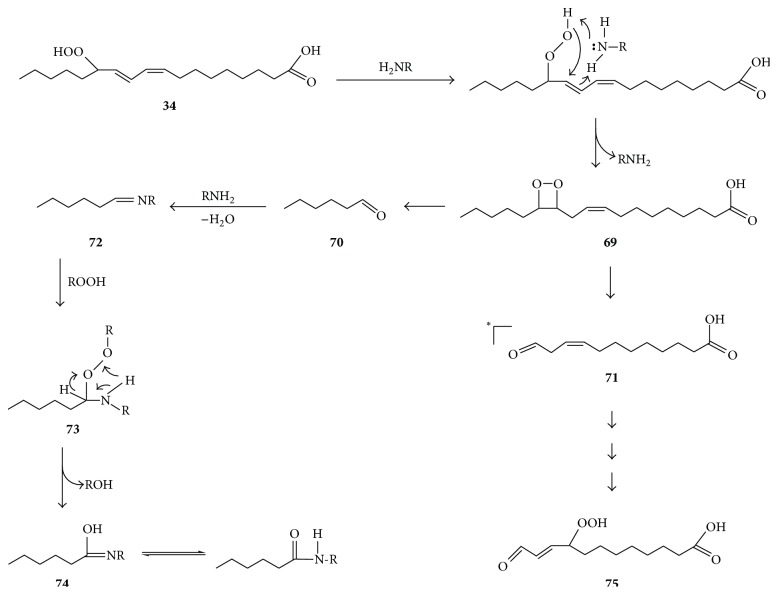
Proposed mechanism for the reaction of the 13-hydroperoxide of linoleic acid** 34** with lysine (RNH_2_) to form N^*ε*^–(hexanoyl) lysine** 74** via a dioxetane intermediate** 69**. The asterisk indicates that the carbonyl is in excited state.

Organic hydroperoxides (ROOH) may participate in singlet oxygen generation through reactions related to Figures 11 and 12. For example, Kato et al. [89] reported that the 13-hydroperoxide of linoleic acid (13-hydroperoxy-9, 11-octadecadienoic acid, and HPODE) reacts with lysine to form N^*ε*^-(hexanoyl) lysine and that this product is not formed by reaction of preformed aldehyde with lysine in the absence of the hydroperoxide. However, the exact mechanism of formation of this product, which is regarded to be proatherogenic and a marker of lipid hydroperoxide-derived modifications of biomolecules [89], has not been elucidated. As suggested in Figure 14, formation of this adduct may begin with lysine-catalyzed conversion of HPODE** 34** to a dioxetane** 69**, whose decomposition affords hexanal** 70** and 12-oxo-9-dodecenoic acid** 71**, and one of these aldehydes may be in an excited state and thus contribute to singlet oxygen formation via energy transfer to triplet oxygen. Subsequent reaction of hexanal** 70** with lysine (RNH_2_) affords corresponding Schiff's base** 72**, whose reaction with another HPODE molecule affords peroxide** 73** which decomposes to form an alcohol and hexanoyl-lysine** 74**. Formation of hexanoyl-lysine in this manner is analogous to the formation of formyl lysine in the reaction system consisting of formaldehyde, hydrogen peroxide, and lysine (vide supra, [68]). 12-oxo-9-dodecenoic acid** 71** may subsequently undergo oxidation to form 12-oxo-9-hydroperoxy-dodecenoic acid** 75** analogously to the known conversion of 3-nonenal to 4-hydroperoxy-2-nonenal [90]. The possibility that lysine catalyzes formation of dioxetane** 69** suggests that proteins can promote formation of toxic aldehydic lipid oxidation products. It also indirectly supports the feasibility of cyclization of fatty acid peroxyl radicals into dioxetanyl radicals as shown in Figure 8.

**Figure 15:**
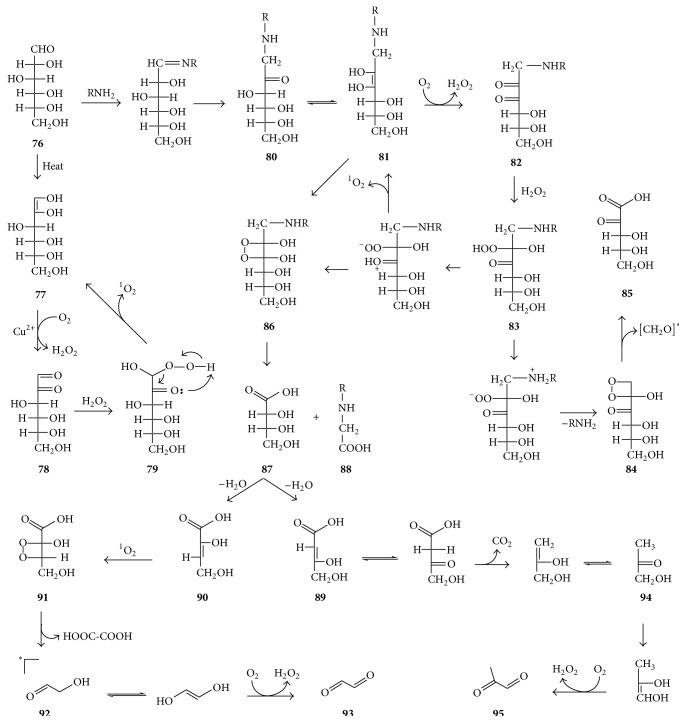
Proposed pathways of formation of ^1^O_2_ and excited carbonyls during Maillard and glycoxidation reactions. The asterisk indicates that the carbonyl is in excited state.

Wang et al. [91] recently found that thermal treatment of pure glucose or fructose solutions up to 70°C led to formation of both hydrogen peroxide and singlet oxygen. Using glucose as an example, the potential mechanism for singlet oxygen generation during heating of such solutions is suggested in Figure 15. It starts with enolization of glucose** 76** to form 1,2-dienol** 77**. Enediols naturally transfer one electron to an oxygen molecule, especially in the presence of oxidized metals such as Cu^2+^, with formation of superoxide anion [92]. Thus, enediol** 77** will be converted to dicarbonyl** 78**, while dismutation of the superoxide anions will produce H_2_O_2_. Subsequent reaction of H_2_O_2_ with dicarbonyl** 78** generates keto-hydroxy-hydroperoxide** 79**, whose decomposition may produce singlet oxygen analogously to the decomposition of tyrosine hydroperoxide** 13** in Figure 5.

The Maillard reaction has also been found to lead to the production of H_2_O_2_ and singlet oxygen both in vitro and in vivo, and the Amadori product was identified as one of the precursors of hydrogen peroxide [93]. As shown in Figure 15, Amadori product** 80** may be converted via enediol** 81** to dicarbonyl** 82**, with coformation of H_2_O_2_. Reaction of the latter two affords hydroxy-hydroperoxide** 83** which may release ^1^O_2_ and convert back to enediol** 81**. Alternatively,** 83** may release lysine and may be converted to dioxetane** 84**, whose decomposition affords ketoacid** 85** and formaldehyde, which may be in the excited state and thus contribute to singlet oxygen formation. Enediol** 81** may also react with singlet oxygen to form dioxetane** 86** whose decomposition produces erythronic acid** 87** and carboxymethyllysine** 88**. The latter is one of the most commonly formed advanced glycation end products(AGEs) that are known to contribute to various physiological disorders [94]. Dehydration of erythronic acid** 87** may produce two regioisomeric deoxy-ketonic acids** 89** and** 90**. The latter may react with singlet oxygen to produce dioxetane** 91** as a precursor of oxalic acid (HOOC-COOH), glycolaldehyde** 92**, and glyoxal** 93**, all of which are known products of glycoxidation [95]. Deoxy-ketonic acid** 89** may, via its ketoform, decarboxylate to form monohydroxyacetone** 94** (via the enol form of the latter), whose oxidation produces methylglyoxal** 95**. Since the formation of the highly reactive glyoxal** 93** and methylglyoxal** 95** is accompanied by H_2_O_2_ formation, singlet oxygen formation according to Figures 10 and 11 will thus also occur during Maillard reaction.

Treatment of cultured U937 human leukemic cells or human multiple myeloma cells with H_2_O_2_ was found to cause singlet oxygen from both the cells and medium components, and this was not dependent on lipid oxidation [47, 96]. The mechanisms in Figures 10, 11, and 15 may be involved in such systems.

## 11. Evidence for Endogenous Ozone Formation and the Potential Mechanisms Involved

Wentworth et al. [7] were the first to suggest the possibility of the formation of ozone (O_3_) in biological systems. One of their key pieces of evidence was that, in solutions of antibodies exposed to singlet oxygen, there was generation of a large amount of hydrogen peroxide, the occurrence of higher bactericidal activity than what could be attributed exclusively to H_2_O_2_, as well as the oxidation of cholesterol to secosterol aldehyde A (**49** in Figure 9), a well-known product of the ozonolysis of cholesterol. They referred to the generation of H_2_O_2_ and ozone under such circumstances as the antibody-catalyzed water oxidation pathway and proposed the idea that this involves an initial reaction of water with ^1^O_2_ to form dihydrogen trioxide (H_2_O_3_) and that decomposition of the latter affords H_2_O_2_ and O_3_ (see equation (7)). This reaction was suggested to occur in a hydrophobic site in the antibody molecule, where the H_2_O_3_ would be shielded from hydrolysis and facilitated to undergo the conversion to H_2_O_2_ and O_3_ [97]. Although antibodies produce much more H_2_O_2_ and O_3_ than other proteins [7], Yamashita et al. [8] reported that antibody catalysis is not essential for this reaction, but rather the presence of one of four amino acids: histidine, tryptophan, cysteine, or methionine. On the other hand, various authors have expressed reservations concerning the generation of ozone under such systems, for example, based on the fact that the catalytic mechanisms for the antibody- or amino acid-catalyzed water oxidation remain ill-defined [1, 3, 35, 37]. Others have reported that cholesterol 5-hydroperoxide** 46**, a product of the oxidation of cholesterol by singlet oxygen, can also decompose to generate secosterol aldehydes [98, 99]. On the other hand, it has been shown that reaction of cholesterol with ozone predominantly generates secosterol A while decomposition of cholesterol-5 hydroperoxide predominantly generates secosterol B [61]. The fact that secosterol A is the predominant secosterol detected in human tissues and is formed by neutrophils in vitro thus supports the formation of endogenous ozone [61, 100]. There is also indirect evidence consistent with the formation of ozone in plant leaves or in the cyanobacterium* Synechocystis* PCC 6803 during light-induced damage to their PS II, because singlet oxygen and tryptophan or histidine residues, respectively, are involved [34]. Unlike other commonly generated ROS that only generate single strand breaks in DNA, ozone generates both single strand and double strand breaks [101, 102]. The addition of L-histidine to cultured mammalian cells exposed to H_2_O_2_ results in DNA double strand breaks [103–105], and this might be related to histidine-mediated ozone generation in the presence of singlet oxygen.

A BioArena system is an overpressured layer chromatography (OPLC) system which enables observation of biochemical interactions between microorganisms and biologically active compounds in an adsorbent layer covered with the microorganisms [106]. Using such a system, it was found that formaldehyde, singlet oxygen, and ozone are formed in the interaction between microorganisms and antibiotic substances such as resveratrol and cinnamic acid and that ozone-trapping compounds greatly reduce the antimicrobial effect [6, 69, 71, 72]. Based on results from the BioArena system, ozone is considered to be an indispensable endogenous molecule that can be detected and measured in practically all biological systems [6, 71, 72]. Moreover, in directly detecting ozone formation by plant leaves through GC-MS-SIM, Balla and Tyihák [4] have added direct proof for the formation of ozone in biological systems. Thus, the current evidence for endogenous ozone is enough to warrant further studies on the mechanisms of its formation and biological effects.

**Figure 16:**
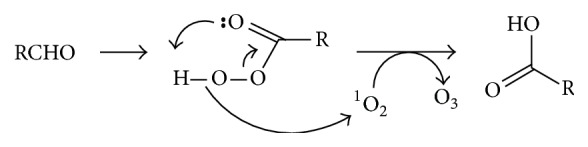
Mechanism of O_3_ formation by ^1^O_2_-mediated deoxidation of a peroxyacid [34].

As already mentioned, a number of questions remain unanswered regarding the antibody-/amino acid-catalyzed water oxidation pathway for ozone generation. Moreover, decomposition of dihydrogen trioxide (H_2_O_3_), the proposed key precursor of ozone in both aqueous and organic solvents, has only been shown to produce singlet oxygen and water rather than hydrogen peroxide and ozone [107]. In response to these challenges, a new concept for ozone formation was recently suggested, involving the oxidation of organic substrates such as aldehydes or amino acids to form oxidized intermediates, and the subsequent singlet oxygen-mediated deoxidation of the oxidized intermediates to produce ozone, whose subsequent decomposition in water affords hydrogen peroxide [34]. For example, an aldehyde (RCHO) may be converted to a peroxyacid [RC(O)OOH] through a radical pathway [48] or by reacting with singlet oxygen [108], and the peroxyacid may undergo a Bayer-Villiger type reaction with singlet oxygen to produce an acid and O_3_ (Figure 16) [34]. The significance of this mechanism is that it might generate ozone in many of the situations where singlet oxygen is formed in the presence of aldehydic compounds such as in Figures 10–15. Detailed potential mechanisms for histidine-, methionine-, and tryptophan-mediated ozone production have been proposed [34]. As an example, part of the reaction scheme for the tryptophan-dependent ozone generation is given in Figure 7. Thus, ^1^O_2_, acting as an electrophile, deoxidizes zwitterion** 23** and alkoxide** 26**, resulting in formation of two O_3_ molecules and regeneration of tryptophan.

## 12. The Significance of Endogenous Singlet Oxygen and Ozone in Human Health

Harman [109] proposed the free radical theory of aging (FRTA) that considers free radical-induced damage to key biomolecules such as DNA and proteins as having a causative role in aging and reduced lifespan. A modified version of the FRTA is the mitochondrial free radical theory of aging (MFRTA), which considers the mitochondrion as the primary source and target of the damaging free radicals [110]. Formation of reactive oxygen species (ROS) from the electron transport chain (ECT) is the basis of the FRTA and MFRTA [111].

As high as 1–5% of consumed oxygen may be converted to superoxide anions, which are readily converted to H_2_O_2_, and the latter is the principal mediator of cellular oxidative stress [111–113]. Reactive nitrogen species are also formed in the mitochondrion, since there is facile diffusion of nitric oxide (NO) to this organelle [114], and the presence of nitric oxide synthases has been demonstrated in mitochondria from various tissues in rats or mice [115, 116] as well as in cultured human cells [117]. In mitochondria, NO increases the formation of superoxide anions and H_2_O_2_ [118]. Thus, there exists suitable conditions in the mitochondria for the diffusion-controlled reaction of NO and superoxide anions to form peroxynitrite (see equation (4)) [114, 119] and subsequent reaction of the latter with H_2_O_2_ to generate singlet oxygen (see equation (5)) as one of the mitochondrial ROS. Peroxynitrite also initiates lipid oxidation [120] and may thus lead to further formation of singlet oxygen in the mitochondria by the Russel mechanism, or activation of lipid-derived carbonyls by cytochrome c oxidase, or the reaction of hydrogen peroxide with such carbonyls. In agreement with the operation of such mechanisms of singlet oxygen formation in the mitochondria, the superoxide anion-dependent formation of this ROS was demonstrated in mitochondria of rat liver and small intestine [121]. Additionally, Berneburg et al. [122] found that exposing normal human fibroblasts to sublethal doses of UVA led to singlet oxygen-dependent deletion of a 4,977-base pair in mitochondrial DNA, which is a common mutation associated with photoaging of human skin. The major product of mitochondrial DNA oxidation is 7,8-dihydro-8-oxoguanine (8-oxoG) [111, 123], and singlet oxygen contributes predominantly to the formation of this compound in DNA [124]. Although the reaction of hydroxyl radical with DNA also produces some 8-oxoG, this is a minor reaction [124, 125]. Thus, singlet oxygen should be an important contributor to aging according to the MFRTA. Besides, generation of singlet oxygen in the presence of carbonyls, amino acids, and proteins in the mitochondrion provides an environment for the generation of mitochondrial ozone, which might contribute to mitochondrial DNA double strand breaks.

The MFRTA has been used to explain the fact that dietary supplementation with antioxidants has not clearly shown antiaging effects, in that the dietary antioxidants may not effectively reach the mitochondria, the main sites of ROS generation, and age-related damage [110, 126]. In support of this, mice overexpressing mitochondria-targeted catalase were found to have improved lifespan, delayed cardiac pathology, and delayed cataract development [126, 127]. The beneficial effects of mitochondria-directed catalase might in part be due to reduced singlet oxygen and ozone, since H_2_O_2_ participates in a good number of the singlet oxygen-generating pathways (*vide supra*).

Another concept that is currently gaining ground is that of mitochondrial hormesis or mitohormesis, which suggests that low levels of reactive oxygen species such as superoxide anions are in fact part of normal physiology and are beneficial for longevity and metabolic health [126, 128–130]. The essence of this is that, at low levels, ROS act as signaling molecules that promote resistance to oxidative stress through increased endogenous antioxidant defense [126]. Hence, effects of ROS may be biphasic, whereby low levels are considered to be beneficial while high levels are detrimental [129]. Such an effect was demonstrated by treatment of elderly patients with a small amount of ozone by rectal sufflation, which resulted in improved antioxidant status and reduced biomarkers of lipid and protein oxidation [131]. A similar treatment was found to reduce the glycemic index and oxidative stress in diabetic patients [132]. Such beneficial effects of ozone therapy were suggested to be a result of the moderate oxidative stress under such conditions activating the nuclear factor-erythroid 2-related factor 2 (Nrf2), which then induces transcription of the antioxidant response elements (ARE), resulting in the production of numerous antioxidant enzymes including catalase as well as phase II detoxification enzymes and heat shock proteins [133]. However, more work is needed to determine the boundaries between the beneficial levels of ROS and the deleterious levels that promote physiological disorders.

As already mentioned, singlet oxygen generated photodynamically in the skin contributes to photoaging of the skin, and this at least partly occurs as follows: ^1^O_2_ reacts with cholesterol to form cholesterol-5-hydroperoxide, which induces expression of matrix metalloproteinase-9 (MMP-9), which in turn degrades collagen and thereby induces the formation of wrinkles and sagging [2, 22]. Singlet oxygen also induces photoaging-associated mutations in mitochondrial DNA [122]. Porphyrias are rare diseases involving a disorder in heme synthesis and are manifested by accumulation of porphyrins in tissues [134]. During cutaneous porphyrias, there is enhanced photosensitivity due to singlet oxygen formation [134].

Singlet oxygen-specific linoleic acid oxidation products, 10-hydroperoxy-8(E), 12(Z)-octadecadienoic acid (10-(E,Z)-HPODE), and 12-hydroperoxy-9(Z), 13(E)-octadecadienoic acid (12-(Z,E)-HPODE) have been found to be suitable biomarkers for the evaluation of the early stages of diabetes, underscoring the role of singlet oxygen in the pathogenesis of this disorder [135, 136]. Further, fasting levels of 10-(E,Z-HPODE) and 12-(Z,E)-HPODE, together with insulin and leptin/adiponectin, are excellent predictors of the risk for type II diabetes, glucose intolerance, and insulin resistance [137, 138]. Patients with diabetes are prone to other diseases and physiological disorders including atherosclerosis, cardiovascular diseases, chronic kidney disease, retinopathy, and skin disorders [139]. Therefore, by contributing to glucose intolerance, insulin resistance, and type II diabetes, singlet oxygen may be contributing to a much wider range of chronic diseases.

Recently, the catabolism of polyamines has been linked to the development of some cancers such as gastric cancer, prostate cancer, and colon cancer [84, 85, 140]. Human spermine oxidase isoforms were found to be localized in the nucleus where they generate reactive oxygen species close to DNA and nuclear proteins [85]. Spermine oxidase-catalyzed catabolism of spermine thus generates products that can react to produce singlet oxygen and ozone in the nucleus. Hence, singlet oxygen and ozone might play a key role in spermine oxidase-dependent cancers.

Since singlet oxygen is a precursor of endogenous ozone, some of the biological activities attributed to singlet oxygen might be directly mediated by ozone or ozonolysis products. High levels of cholesterol secosterols A and B have been detected in human atherosclerotic tissues and shown to be proatherogenic [141]. The fact that secosterol A is the predominant secosterol in human tissues [61, 62, 100] indicates a much greater role for ozone than singlet oxygen in the in vivo formation of the secosterols because even the secosterol B detected in such systems is at least partly formed by aldolization of secosterol A. A possible reason for the in vivo predominance of secosterol A is that, while cholesterol hydroperoxide-5, a direct precursor of secosterol B, may be easily formed in tissues, its Hock cleavage is favored by acidic conditions, which may not be common in most tissues. The fact that Tomono et al. [142] observed a time-dependent increase of the secosterols in plasma of mice after injection of lipopolysaccharide indicates that ozone formation is easily initiated during inflammation.

The secosterols A and B induce endothelial cell dysfunction by promoting apoptosis and inhibiting endothelial-dependent arterial relaxation [143]. These products are elevated in brain tissues of Alzheimer's disease patients, and it was demonstrated that they may trigger the disease by inducing protein misfolding [144]. Elevated levels of the secosterols are found in Lewy body dementia brain tissues, where they accelerate alpha-synuclein fibrillization [145]. The secosterols bind to the p53 protein and induce it to misfold and lose the ability to bind to a consensus DNA sequence, signifying that they may contribute to cancers where wild-type inactivation of p53 occurs, such as breast cancer, colon cancer, colon adenoma, and neuroblastoma [146]. The secosterols and their acidic oxidation products are strongly cytotoxic against various human cell lines, and it was concluded that, during inflammation, cell death caused by them may contribute to further tissue damage and development of disease [147]. Formation of Schiff's bases between the secosterols and myelin basic protein (MBP) induces myelin instability and might contribute to the onset and progression of multiple sclerosis [148].

Despite the generally harmful effects of high levels of ROS, organisms employ high ROS levels in certain pathological situations in an attempt to restore normal physiological conditions. For example, singlet oxygen and ozone are produced by activated human neutrophils, where they are important for the destruction of bacteria during acute infections [7, 149]. The use of photodynamic therapy (PDT) in cancer treatment relies on the generation of singlet oxygen in the tissues to destroy cancer cells, and when PDT involves use of sensitizers conjugated to antibodies, ozone generation might be equally important [34].

## 13. The Influence of Dietary Factors on the Formation and Effects of Endogenous Singlet Oxygen and Ozone

Some dietary factors promote inflammation and oxidative stress, while others are anti-inflammatory and protective against oxidative stress. Generally, the proinflammatory factors may promote while the anti-inflammatory factors may inhibit the formation of singlet oxygen and ozone. More specifically, some of the dietary factors, such as carotenoids, act as quenchers of singlet oxygen. Such substances will prevent ozone formation and dysfunctions associated with singlet oxygen. Dietary carotenoids have been shown to easily accumulate in the skin, where they prevent the singlet oxygen-associated photoaging [2, 22, 150]. Just as singlet oxygen plays a role in the development of diabetes (vide supra), adequate dietary carotenoids have been reported to reduce the risk for type II diabetes and the metabolic syndrome [151–153]. Besides their singlet oxygen-quenching ability, carotenoids also have anti-inflammatory activity by interfering with the NF-*κ*B pathway [154]. Beta carotene was reported to prevent ozone-induced proinflammatory markers in murine skin [155].

On the other hand, alcohol consumption lowers the levels of carotenoids in the human skin and increases the risk for sunburn and potentially skin cancer [156]. Alcohol consumption and cigarette smoking also lower serum carotenoid levels [157]. By lowering carotenoids, alcohol consumption and cigarette smoking reduce singlet oxygen quenching and may promote ozone formation. Moreover, since ethanol is metabolized to acetaldehyde, there are increased blood levels of acetaldehyde after alcohol consumption [158] and this may promote production of ROS. Alcohol consumption is also associated with oroesophageal squamous cell carcinoma, gastric cancer, and colorectal cancer, which may be partly due to induction of ROS production by ethanol or its metabolite, acetaldehyde [159–161].

*Helicobacter* strains expressing the virulence factor cytotoxin-associated gene A (Cag-A) stimulate the expression of spermine oxidase in gastric epithelial cells and promote ROS generation and DNA damage in these cells, thereby increasing gastric cancer risk [83]. SMO expression also contributes to ulcerative colitis, an inflammatory condition that is associated with colon cancer [162]. The bacterium* Enterococcus faecalis* produces large amounts of superoxide anions and hydrogen peroxide in the colon and this causes DNA damage in luminal cells of the colon [163]. The large amounts of H_2_O_2_ may promote ^1^O_2_ and O_3_ formation in that environment. In addition, some colon microorganisms convert bile components into metabolites that are carcinogenic, partly by inducing ROS generation [164]. Consumption of foods containing probiotic microorganisms may reduce oxidative stress in various ways including inhibiting the growth of oxidative stress-inducing microorganisms [164, 165].

High fat and high carbohydrate diets are associated with increased postprandial inflammation and oxidative stress [166]. Hence, such diets may promote the generation of singlet oxygen and ozone, besides other ROS. The consumption of foods rich in Maillard oxidation products may also contribute to endogenous singlet oxygen and ozone generation because dietary advanced glycation end products promote oxidative stress and inflammation through interaction with the receptor for advanced glycation products in a variety of cells, resulting in oxidative stress involving superoxide anion and H_2_O_2_ formation [167–169].

Various dietary phenolic substances are recognized as powerful antioxidants mainly by their scavenging of free radicals. On the other hand, these phenolic substances also have prooxidant activity in that they can react with oxygen to generate superoxide anions, hydrogen peroxide, and singlet oxygen [31, 52, 53]. Thus, consumption of coffee or tea, which is rich in these phytochemicals, also means consumption of substantial amounts of hydrogen peroxide and perhaps singlet oxygen [73, 74]. Nevertheless, the consumption of foods rich in such substances, such as fruits and vegetables, and even tea and coffee has mainly been associated with beneficial effects [170–172]. This is because, apart from direct antioxidant or prooxidant activity, these compounds also affect cellular physiology by signaling pathways. For example, they may inhibit redox-sensitive transcription factors and prooxidant enzymes such as xanthine oxidase or nitric oxide synthase [171] and/or induce expression of antioxidant enzymes and phase II detoxification enzymes [170–172]. Phytochemicals in virgin olive oil were found to lower postprandial inflammation by reducing postprandial plasma lipopolysaccharide levels [173].

## 14. Conclusion

Singlet oxygen may be commonly generated in tissues through a range of enzymatic and nonenzymatic reactions, and, at least based on the in vivo formation of cholesterol secosterol aldehydes, ozone formation also seems to be important. Endogenous overproduction of these two oxidants likely plays important roles in the pathogenesis of physiological disorders such as diabetes, cardiovascular diseases, skin photoaging, and some cancers. Consumption of foods rich in singlet oxygen quenchers and components with anti-inflammatory activities, including probiotics, may help reduce the negative effects of high levels of these oxidants. Phytochemicals that generate low levels of these oxidants might also be useful for cellular adaptation to oxidative stress and prevention of physiological disorders.
